# Research on the Mechanism of Cognitive Decline in Patients With Acoustic Neuroma

**DOI:** 10.3389/fnins.2022.933825

**Published:** 2022-07-04

**Authors:** Xueyun Deng, Lizhen Liu, Jun Luo, Lihua Liu, Xuhui Hui, Hua Feng

**Affiliations:** ^1^Department of Neurosurgery, Southwest Hospital, Third Military Medical University, Army Medical University, Chongqing, China; ^2^Department of Neurosurgery, The Affiliated Nanchong Central Hospital of North Sichuan Medical College, Nanchong, China; ^3^Department of Radiology, Southwest Hospital, Third Military Medical University, Army Medical University, Chongqing, China; ^4^Department of Geriatrics, The Affiliated Nanchong Central Hospital of North Sichuan Medical College, Nanchong, China; ^5^Department of Neurosurgery, West China Hospital, Sichuan University, Chengdu, China

**Keywords:** cognition, acoustic neuroma, vestibular schwannoma, diffusion tensor imaging, tract-based spatial statistics (TBSS)

## Abstract

Little is known about neuropsychological research on patients with acoustic neuroma (AN), especially cognitive neuropsychology. We aim to compare the cognitive function of patients with AN and healthy controls (HCs) and explore possible underlying mechanisms. Various neuropsychological assessments were performed on all participants. Tract-based spatial statistics (TBSS) was used to compare DTI metrics such as fractional anisotropy (FA), axial diffusivity (AD), radial diffusivity (RD), and mean diffusivity (MD). Correlation analysis was analyzed between DTI metrics and cognitive scales. Compared with the HC group, the AN group performed worse in the neuropsychological evaluations, and TBSS analysis showed widespread alteration of the FA, AD, RD, and MD, which correlated with the cognitive function. These white matter tracts include minor forceps, major forceps, anterior thalamic radiation, superior longitudinal fasciculus, corticospinal tract, and right inferior fronto-occipital fasciculus. Meanwhile, we found for the first time that cognitive decline was related to the decrease of FA in minor forceps, which can be used as a neurobiological marker of cognitive impairment in patients with AN. The occurrence of cognition impairment is common in patients with AN. Including neuropsychological evaluation in the routine clinical assessment and appropriate treatment may strengthen clinical management and improve the quality of life of patients.

## Introduction

Previous research has demonstrated that patients with bilateral deafness are associated with cognitive impairment. Cognition decline in elderly people with severe and moderate bilateral hearing loss is 1.6 and 1.4 times higher than that in a normal person, respectively (Davies et al., 2017). Presbycusis is more likely to develop cognitive impairment, such as memory decline (Lin et al., 2011), psychomotor processing disorders (Chen et al., 2018), and decreased executive function (Gurgel et al., 2014). For patients with bilateral presbycusis, early hearing intervention can improve the behavior state and cognition function (Ma et al., 2016; Adrait et al., 2017). Research confirms that unilateral hearing loss (UHL) can also affect cognitive function. UHL children develop worse cognitive performance and poorer language than normal children (Lieu et al., 2012; van Wieringen et al., 2019). The brain network connections, such as language, executive function, and cognition, are altered in children with UHL (Jung et al., 2017).

Acoustic neuroma (AN), often called vestibular schwannoma, is an important contributor to UHL. AN is a benign tumor, which originates from the VIII cranial nerve sheath (Evans et al., 2005). AN accounts for around 6% of all intracranial tumors, and 85% of the tumors in the cerebellopontine angle region (Fortnum et al., 2009). About 90% of patients with AN perform ipsilateral side UHL (Suzuki et al., 2010). UHL is defined as hearing loss in one ear and normal in the other one (Vincent et al., 2015). UHL can cause a decline in the ability of sound source localization and communication in a noisy environment (Nelson et al., 2019). Some studies have demonstrated that cognitive decline occurs in patients with AN. Goebel and Mehdorn (2018) included 45 patients (AN: meningiomas = 27:18) and found that 69% of patients present cognitive impairment. And the most common cognitive performance is visual movement speed and attention (alertness). Fan et al. (2020) demonstrated that cognitive decline has been observed in some patients with AN. To date, there are few reports on whether AN will cause cognitive dysfunction, and the number of reported cases is small. More importantly, little is known about the mechanism. In our study, first, using various neuropsychological tests, we compared the cognition between AN and healthy control (HC) groups. Then we explore the possible mechanism using the tract-based spatial statistics (TBSS) method based on diffusion tensor imaging (DTI) data. DTI takes advantage of the anisotropy of diffusion of water molecules in different tissues to measure the dispersion motion of water molecules and can be used to evaluate the fiber direction, density, integrity of myelin sheath, and morphology of axons. The DTI metrics most commonly include fractional anisotropy (FA), axial diffusivity (AD), radial diffusivity (RD), and mean diffusivity (MD).

In patients with UHL, some findings are obtained using DTI. Wu et al. (2009) used region of interest (ROI) to measure FA, AD, and RD in the lateral lemniscus and inferior colliculus of 19 patients with long-term unilateral deafness. The authors found that the FA value decreased, indicating demyelination or axonal injury in the lateral lemniscus and inferior colliculus. Rachakonda et al. (2014) found that in addition to the decrease of FA in the left lateral lemniscus, changes in other non-auditory pathways, for example, the microstructural integrity was affected in the superior temporal gyrus and middle cerebellar peduncle in 29 children with UHL (7–17 years old). Vos et al. (2015) found that FA decreases significantly in both hemispheres of patients with UHL. At present, there are few studies on the white matter fiber of UHL and even less on the relationship between UHL and cognitive function. Therefore, we design this experiment to study the changes in white matter fiber metrics (FA, MD, AD, and RD) in the whole brain based on TBSS and aim at finding the relationship between DTI metrics and cognitive function.

## Materials and Methods

### Participants

In total, 69 right-handed patients with AN (mean age: 50.3 ± 13.1 years, age range: 19–76 years, and 44 women) who were recruited from Neurosurgery of West China Hospital between October 2019 and July 2020 underwent neuropsychological evaluations and DTI scanning. The median years of education of patients with AN were 9.62 years, and the interquartile range was 6.50 years. The median course of disease of patients with AN was 2.0 years, interquartile range was 3.6 years. Acoustic neuroma was subdivided into left acoustic neuroma (LAN) and right acoustic neuroma (RAN). A total of 44 patients with LAN (mean age: 50.3 ± 13.6 years, age range: 20–76 years, 25 women, median years of education: 11.0 years, and interquartile range: 7.3 years) and 25 patients with RAN (mean age: 50.3 ± 12.5 years, age range: 19–73 years, 19 women, median years of education: 9.0 years, and interquartile range: 9.0 years) received hearing test and tinnitus handicap inventory if present tinnitus symptom. Seventy right-handed HCs (mean age: 46.5 ± 10.1 years, age range: 26–74 years, 48 women, median years of education: 9.0 years, and interquartile range: 7.0 years) were enrolled in our study, with age, gender, and years of education matched to the patients. The HCs underwent the same neuropsychological evaluations and DTI scanning. All participants reported no previous or current psychiatric disorders. The experiment was approved by the hospital ethics committee, and all participants signed the informed consent.

### Clinical Assessment and Neuropsychological Evaluation

Pure tone average (PTA) was defined as the average pure tone threshold at 0.5, 1, 2, and 4 kHz. According to the World Health Organization (1991), hearing loss was classified as normal hearing (PTA < 25 dB HL), mild loss (PTA: 26–40 dB HL), moderate loss (PTA: 41–60 dB HL), severe loss (PTA: 61–80 dB HL), and profound loss (PTA > 81 dB HL). Based on tumor size, according to Koos classification (Erickson et al., 2019), patients were graded into four groups: grade I (small intrameatal tumor), grade II (intrameatal and extrameatal; no contact with the brainstem), grade III (touching but without compression of the brainstem), and grade IV (large tumor touching the brainstem with compression of the brainstem). The size of LAN was 3.02 ± 1.21 cm, and the size of RAN was 3.06 ± 1.13 cm. No statistical significance between the two groups was detected.

Patients with AN are often complicated with tinnitus. In our study, patients with AN accompanying tinnitus were evaluated using the tinnitus handicap inventory (THI) (Newman et al., 1996). Higher scores reveal greater severity and impact on daily life.

The neuropsychological evaluation for all participants consisted of the evaluation of attention, memory, visuospatial executive, language, orientation, executive control, motor speed, calculation, and abstract. The neuropsychological evaluation included the following measures: Montreal cognitive assessment (MoCA), Rey auditory verbal learning test (RAVLT), Stroop color-word test (SCWT), symbol digit modalities test (SDMT), trail-making test (TMT), Hamilton depression scale (HAMD), and Hamilton anxiety scale (HAMA). MoCA reflects the general cognitive function, including 8 domains (attention, memory, language, orientation, calculation, executive function, abstract, and visuospatial function). RAVLT is used to assess auditory vocabulary learning ability and memory. SCWTs evaluate executive function and attention. SDMT is mainly used to evaluate the speed of information processing, attention, working memory, movement speed, and visual perception ability. TMTs assess attention, executive control, and visuospatial abilities.

### MRI Data Acquisition

Magnetic resonance imaging (MRI) data were performed using a 3.0 Tesla magnet (General Electric Medical System, Milwaukee, WI, USA) and a 32-channel head coil. The DTI data included one *b* = 0 s/mm^2^ and 51 gradient directions using *b* = 1,000 s/mm^2^. The repetition time was 7.0 s and the echo time was 72 ms. A total of 34 slices of 4 mm thickness without a gap, not including the cerebellum, were acquired with matrix size 256 × 256. The flip angle was 90°. The field of view was 24 × 24 cm. The scanning time was about 8 min and 3 s. T1 scanning parameters were as follows: slice thickness was 1 mm, scanning matrix was 512 × 512, and voxel size was 0.5 × 0.5 × 1.0 mm^3^. The scanning time was about 4 min and 37 s. During scanning, earmuffs and earplugs were used to reduce scanning noise. Imagining data were obtained with the same radiological and MR equipment.

## Data Preprocessing

### TBSS Pre-processing

Diffusion tensor imaging (DTI) data processing was performed by FSL software (Jenkinson et al., 2012). The main steps of preprocessing included eddy current correction, obtaining a brain mask, and diffusion tensor estimation. Eddy current correction can correct the deformation caused by head movement to some extent. Adjusted the gradient direction according to the results of eddy current correction. Then the extra-brain image of the b0 image was removed by the bet2 command to reduce invalid calculations, and a mask was obtained. Then the diffusion tensor was estimated using the dtifit function of FSL, and the related metrics such as FA, MD, AD, and RD were obtained at the same.

The metrics were processed through the TBSS software package of FSL. The TBSS processing flow was as follows (Zhang et al., 2020): (1) The individual FA images were registered to the FMRIB58_FA template. (2) The average FA map and white matter skeleton were constructed. (3) Binary white matter skeleton mask was created at a threshold of FA > 0.2. (4) The individual participant's FA was projected onto the FA skeleton. (5) Voxel-level statistical analysis was performed. Using a non-parametric test between groups, the brain regions with statistical differences were obtained, and the random procedures were repeated 5,000 times. (6) Expand the results to show the results better. Differential MD, AD, and RD were obtained by the same methods. The results were corrected by multiple comparisons, using *p* < 0.05, based on the threshold-free cluster enhancement (TFCE) method (Smith and Nichols, 2009). JHU ICBM-DTI-81 White-Matter Labels atlas (Wakana et al., 2004) was used to determine the anatomical location information of differential brain regions. Finally, Spearman's correlation analysis was performed between DTI metrics of the significant cluster as identified by TBSS and cognitive function. The significance level was *p* < 0.05.

### Network Construction and Rich-Club Analyze

The network construction was performed using PANDA (www.nitrc.org/projects/panda) (Cui et al., 2013). The main steps were as follows: data quality check, data format conversion, and head eddy-current effect correction. The whole-brain fiber bundle was tracked using deterministic fiber tracking (Mori et al., 1999). The main results tracked by PANDA software were fiber number (FN). The FN was registered to the individual AAL90 brain region. Due to tumors compressing the cerebellum and brainstem in some patients, it may affect the study of the cerebellum, so we constructed networks based on the 90 cerebral regions of the AAL atlas, excluding the cerebellar regions. The FN value of every two brain regions constructed a weighted matrix. The connections were considered effectively structurally connected if at least three fibers in two brain regions in 80% of the subjects (Shu et al., 2015), transforming the weighting matrix into a binary matrix. Network topological property (degree centrality) was calculated using the GRETNA toolbox (http://www.nitrc.org/projects/gretna/) (Collin et al., 2014) (see Supplementary Table 1).

Sorting by degree centralities from large to small, the first 12% were taken as rich club nodes (Collin et al., 2014). All nodes were divided into rich club nodes and non-rich club nodes. The connections between rich club nodes were defined as rich club connections, the connections between rich club and non-rich club nodes were defined as feeder connections, and the connections between non-rich club nodes were defined as local connections (Collin et al., 2017). The schematic diagram is shown in **Figure 5A**. Rich-club refers to the connections between hub nodes, which are more closely connected than the connections between non-hub nodes, reflecting the difference in brain operation mode. Generally, rich club nodes are chosen from the group average brain network of healthy people because the disease may change the degree of centrality.

### Statistical Analysis

Statistical analysis was performed using SPSS (version 23.0, IBM, USA). Data are expressed as mean ± SD for normally distributed data or median (interquartile range) for not normally distributed data. Categorical variables were presented as a percentage. The Student's *t*-test (two-tailed) was performed if the variables were satisfied normal distribution and homogeneous variance, and Mann–Whitney U non-parametric test was used if the variables were not satisfied normal distribution and homogeneous variance. Comparisons among the three groups were made using one-way ANOVA or Kruskal–Wallis H test, depending on whether or not the data were satisfied normal distribution and homogeneous variance. The qualitative data were compared by the χ^2^ test. To explore the relationship between clinical data and cognition, Spearman's correlation analysis was performed. *P*-value was set to 0.05.

## Results

### Demographic Characteristics

There were no significant differences in gender, age, and years of education between patients and HCs (*p* > 0.05). No significant differences in the course of the disease, PTA on the affected side, and THI scores between the LAN and RAN groups were attested (*p* > 0.05).

### Comparison of Cognition Function and Correlation Analysis

Compared to the HC group, both groups (LAN and RAN) performed worse in MoCA, RAVLT, SCWT B, C, SDMT, and TMT (*p* < 0.05) (see Table 1). Spearman's correlation analysis showed that the left-sided PTA of patients with LAN was negatively correlated with MoCA subscores on visuospatial executive (*r* = −0.502, *p* = 0.004) and delayed recall (*r* = −0.383, *p* = 0.034), and SDMT (*r* = −0.382, *p* = 0.034), and positively correlated with SCWT A (*r* = 0.423, *p* = 0.018) and SCWT B (*r* = 0.474, *p* = 0.007). In patients with RAN, the right-sided PTA was negatively correlated with MoCA subscores on orientation (*r* = −0.566, *p* = 0.014), RAVLT immediate recall (*r* = −0.519, *p* = 0.027), RAVLT delay recall (*r* = −0.476, *p* = 0.046), and SDMT (*r* = −0.498, *p* = 0.036), while positively correlated with SCWT B (*r* = 0.627, *p* = 0.005) and TMT-A (*r* = 0.492, *p* = 0.038).

**Table 1:** Comparison of clinical data and cognitive function among LAN, RAN, and HC groups.

	**LAN (*n* = 44)**	**RAN (*n* = 25)**	**HC (*n* = 70)**	**H value**	***p-*Value**	***Post-hoc***
MoCA	21.00 (6.00)	20.00 (9.00)	25.50 (4.00)	38.792	<0.001*	RAN < HC LAN < HC
SDMT	39.00 (28.00)	34.00 (34.00)	45.00 (28.00)	10.824	0.004*	RAN < HC LAN < HC
RAVLT (immediate recall)	34.00 (16.00)	31.00 (15.00)	47.00 (19.00)	32.651	<0.001*	RAN < HC LAN < HC
RAVLT (delay recall)	6.00 (5.00)	5.00 (4.00)	9.00 (5.00)	18.441	<0.001*	RAN < HC LAN < HC
SCWT A (s)	32.00 (27.00)	36.00 (16.75)	27.00 (14.50)	5.714	0.057	N/A
SCWT B (s)	49.00 (32.00)	53.00 (24.00)	38.00 (20.00)	15.631	<0.001*	RAN>HC LAN>HC
SCWT C (s)	129.00 (83.00)	131.00 (64.00)	85.50 (53.00)	24.963	<0.001*	RAN>HC LAN>HC
TMT A (s)	52.00 (65.00)	67.00 (63.00)	40.50 (30.00)	18.100	<0.001*	RAN>HC LAN>HC
TMT B (s)	170.00 (193.00)	230.00 (191.00)	104.00 (116.00)	15.320	<0.001*	RAN>HC LAN>HC

p-Values were obtained by Kruskal–Wallis non-parameter test. H values were obtained by Kruskal–Wallis. All data were presented as median (interquartile range). ^*^p < 0.05. LAN, left acoustic neuroma; RAN, right acoustic neuroma; HC, healthy control; N/A, not available.

### Comparison of Cognitive Function Among Patients With Different Grades of AN and HC Group

According to Koos classification, there were 1 patient in grade I, 16 cases in grade II, 17 cases in grade III, and 35 cases in grade IV. Because the number of patients with grade I was too small to analyze statistically, the cognitive functions of patients of other grades were compared with those of the HC group. The results showed that compared with the HC group, the cognitive function of patients with grades II–IV decreased, as listed in Supplementary Tables 1, 2.

### Comparison of Cognitive Function Among Patients With Different Degrees of Hearing Loss and HCs

The left PTA in patients with LAN was 55.73 ± 26.14 dB HL, and the right PTA in patients with RAN was 66.74 ± 34.06 dB HL, and there was no statistical significance between groups. According to WHO grade (1996), the hearing of the affected side (tumor side) of the patients with AN was as follows: normal hearing in 5 cases, mild loss in 12 cases, moderate loss in 9 cases, severe loss in 13 cases, profound loss in 10 cases. Compared with the HC group, the cognitive function of patients with AN having mild to profound hearing loss decreased to various degrees, (see Supplementary Tables 3, 4).

### TBSS Analysis

**Figure 1:**
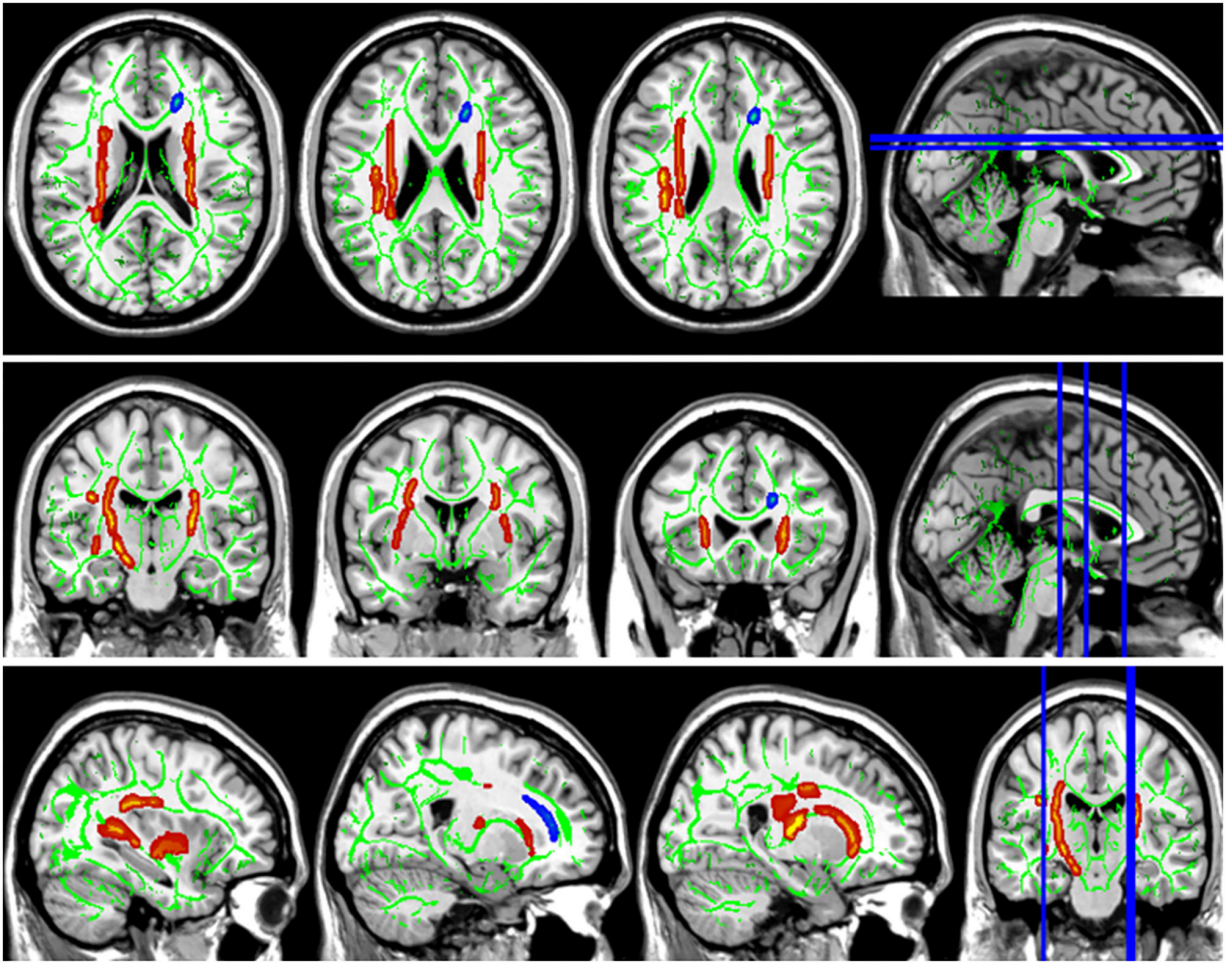
Fractional anisotropy (FA) results of tract-based spatial statistics (TBSS). The background map was constructed by the mean FA skeleton (green) overlaid on the Montreal Neurological Institute (MNI) template. The red voxels represent values increase, and the blue voxels represent a decrease. Group differences in FA. The FA values of left acoustic neuroma (LAN) and right acoustic neuroma (RAN) patients decreased in the minor forceps, while the FA value in the bilateral corticospinal tract and the left superior longitudinal fasciculus increased compared to the healthy control (HC) group.

**Figure 2:**
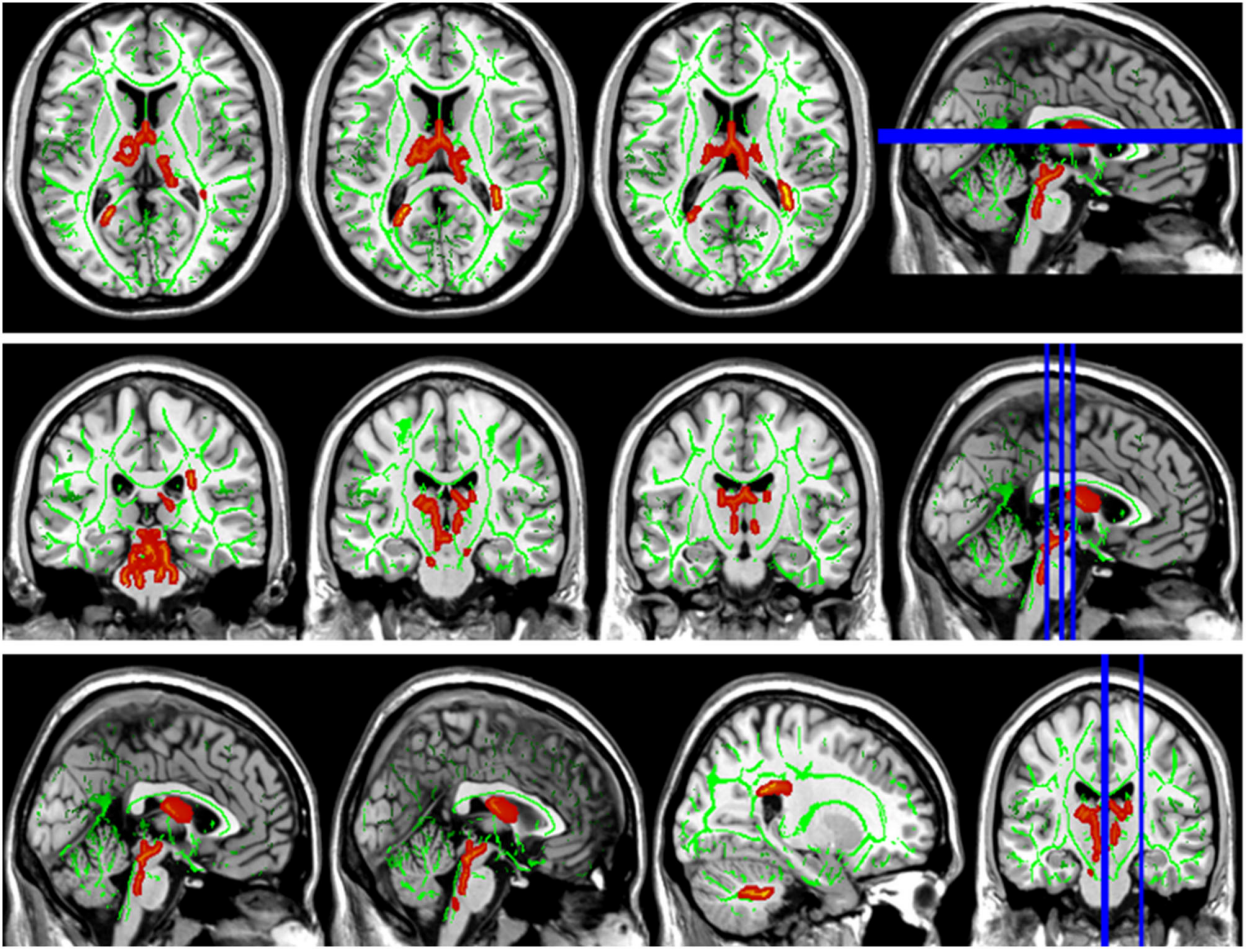
Group differences in mean diffusivity (MD). The MD values in patients with LAN and RAN increased in left anterior thalamic radiation, right inferior fronto-occipital fasciculus, and major forceps.

**Figure 3:**
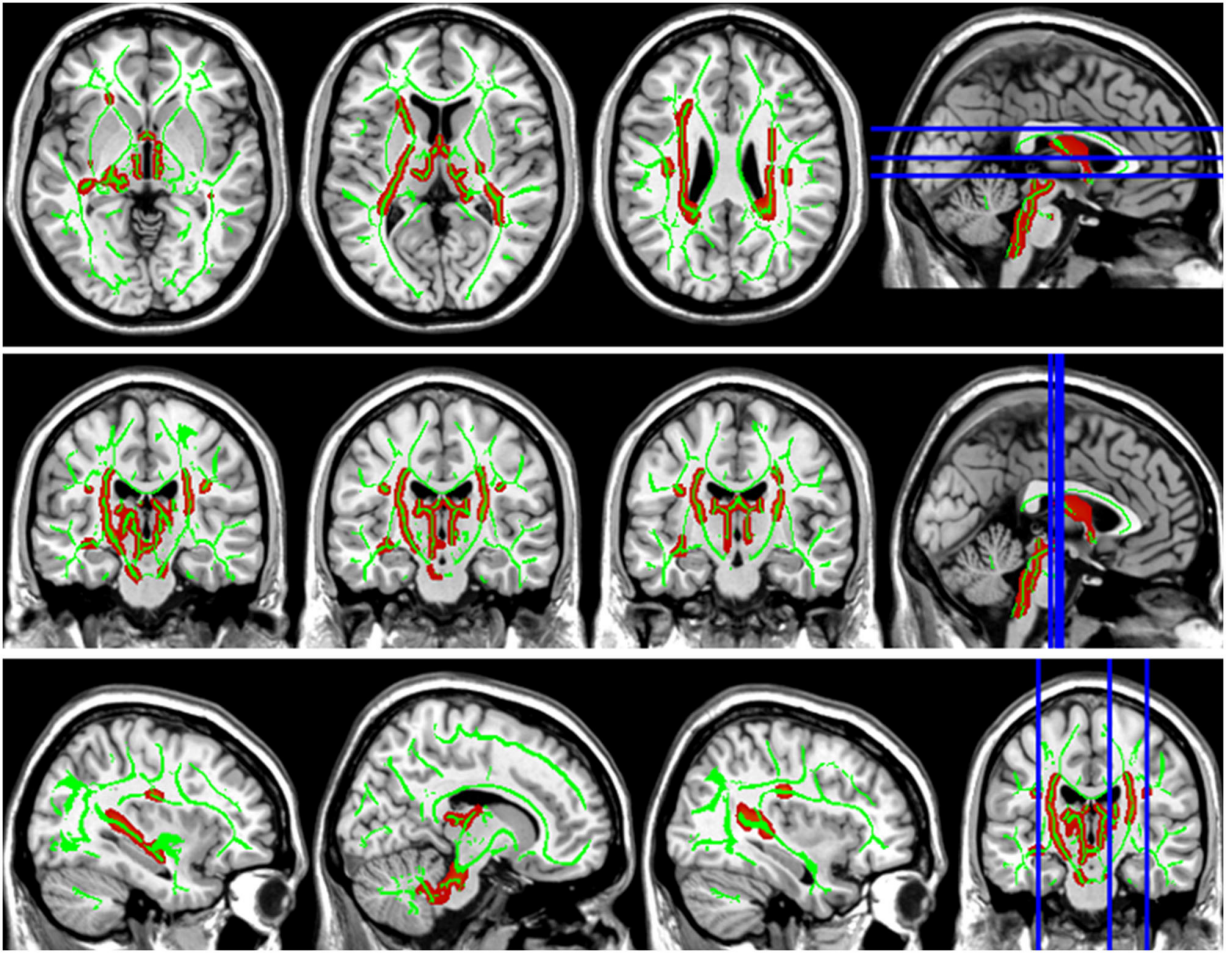
Group differences in axial diffusivity (AD). The AD values in patients with LAN and RAN increased in bilateral superior longitudinal fasciculus, left anterior thalamic radiation, and right corticospinal tract.

**Figure 4:**
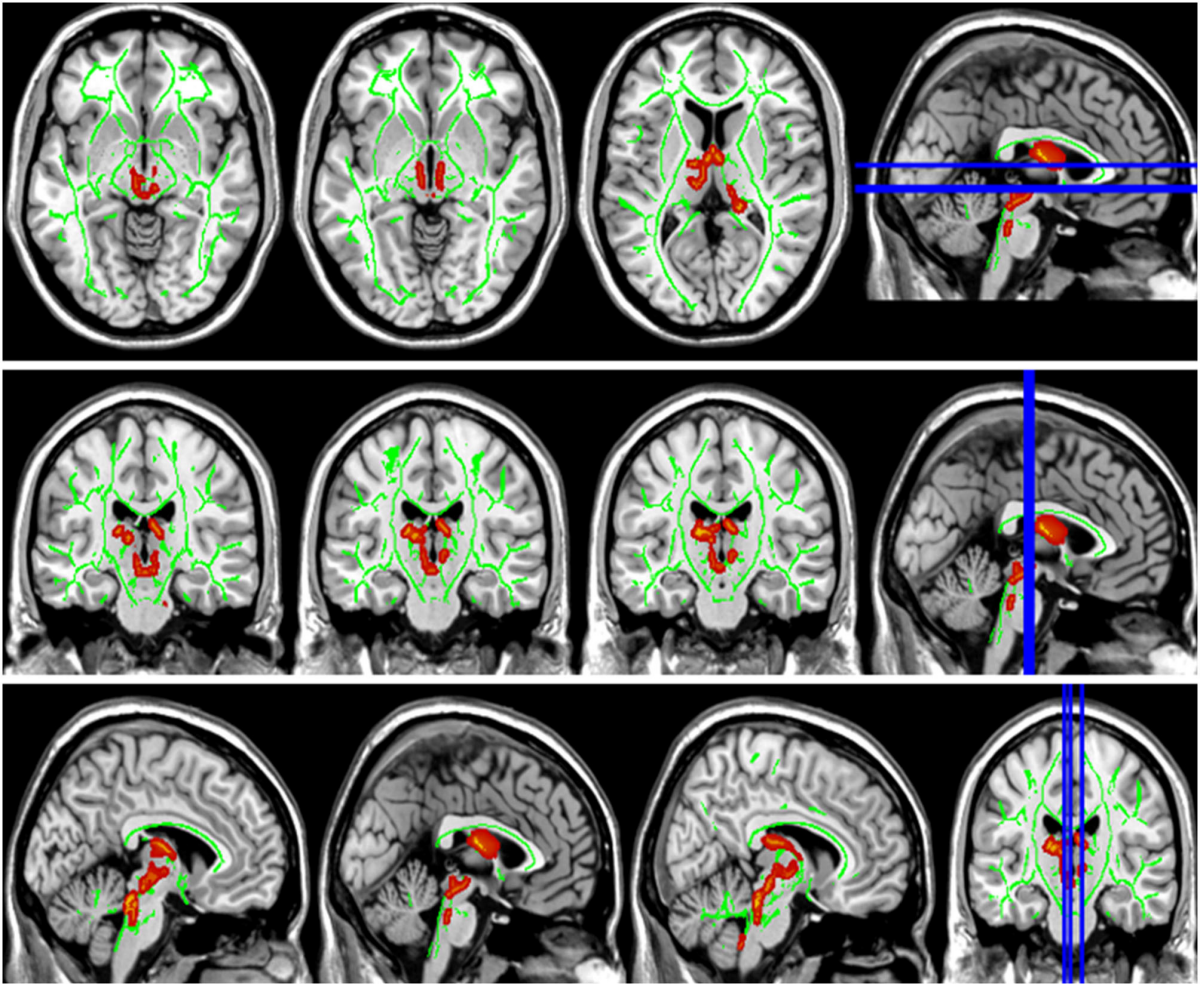
Group differences in radial diffusivity (RD). The RD values in patients with LAN and RAN increased in bilateral anterior thalamic radiation.

Compared with the HC group, the DTI metrics FA, AD, RD, and MD in patients with LAN and RAN changed widely. Compared to the HC group, the FA values in patients with LAN and RAN decreased in the minor forceps, while the FA value in the bilateral corticospinal tract and the left superior longitudinal fasciculus increased (see Figure 1). The MD values in patients with LAN and RAN increased in left anterior thalamic radiation, right inferior fronto-occipital fasciculus, and major forceps (see Figure 2). The AD values in patients with LAN and RAN increased in bilateral superior longitudinal fasciculus, left anterior thalamic radiation, and right corticospinal tract (see Figure 3). The RD values in patients with LAN and RAN increased in bilateral anterior thalamic radiation (see Figure 4). The correlation analysis showed that the changes in these metrics (FA, AD, RD, and MD) mentioned above show an extensive correlation with the neuropsychological variables, which may lead to a cognition decline in patients with AN (see Tables 2, 3).

**Table 2:** Correlation analysis between DTI parameter metrics and cognition scale (1).

	**FA in minor forceps** **(*n* = 139)**	**AD in left anterior thalamic radiation** **(*n* = 139)**	**AD in left superior longitudinal fasciculus** **(*n* = 139)**	**AD in right superior longitudinal fasciculus** **(*n* = 139)**	**AD in right corticospinal tract** **(*n* = 139)**
MoCA scores	0.444 (*p* <0.001)	−0.440 (*p* <0.001)	−0.367 (*p* <0.001)	−0.297 (*p* <0.001)	−0.367 (*p* <0.001)
RAVLT immediate recall	0.368 (*p* <0.001)	−0.473 (*p* <0.001)	−0.242 (0.004)	−0.263 (0.002)	−0.375 (*p* <0.001)
RAVLT delay recall	0.392 (*p* <0.001)	−0.515 (*p* <0.001)	−0.296 (*p* <0.001)	−0.278 (0.001)	−0.413 (*p* <0.001)
SCWT A (s)	−0.341 (*p* <0.001)	0.264 (0.002)	0.284 (0.001)	0.167 (0.054)	0.243 (0.005)
SCWT B (s)	−0.352 (*p* <0.001)	0.351 (*p* <0.001)	0.261 (0.002)	0.177 (0.037)	0.241 (0.004)
SCWT C (s)	−0.377 (*p* <0.001)	0.377 (*p* <0.001)	0.225 (0.008)	0.138 (0.107)	0.256 (0.002)
SDMT	0.446 (*p* <0.001)	−0.368 (*p* <0.001)	−0.272 (0.001)	−0.193 (0.024)	−0.281 (0.001)
TMT A (s)	−0.350 (*p* <0.001)	0.315 (*p* <0.001)	0.287 (0.001)	0.286 (0.001)	0.256 (0.002)
TMT B (s)	−0.364 (*p* <0.001)	0.331 (*p* <0.001)	0.269 (0.001)	0.209 (0.014)	0.260 (0.002)

*Data are presented as Spearman's correlation coefficient (r value) and (p-value). DTI, diffusion tensor imaging; FA, fractional anisotropy; AD, axial diffusivity*.

**Table 3:** Correlation analysis between DTI parameter metrics and cognition scale (2).

	**RD in left anterior thalamic radiation** **(*n* = 139)**	**RD in right anterior thalamic radiation** **(*n* = 139)**	**MD in left anterior thalamic radiation** **(*n* = 139)**	**MD in right inferior frontooccipital fasciculus** **(*n* = 139)**	**MD in major foeceps** **(*n* = 139)**
MoCA scores	−0.336 (*p* <0.001)	−0.391 (*p* <0.001)	−0.392 (*p* <0.001)	−0.368 (*p* <0.001)	−0.404 (*p* <0.001)
RAVLT immediate recall	−0.372 (*p* <0.001)	−0.403 (*p* <0.001)	−0.426 (*p* <0.001)	−0.357 (*p* <0.001)	−0.402 (*p* <0.001)
RAVLT delay recall	−0.402 (*p* <0.001)	−0.416 (*p* <0.001)	−0.451 (*p < * 0.001)	−0.415 (*p* <0.001)	−0.415 (*p* <0.001)
SCWT A (s)	0.225 (0.009)	0.301 (*p* <0.001)	0.237 (0.006)	0.261 (0.002)	0.277 (0.001)
SCWT B (s)	0.321 (*p* <0.001)	0.320 (*p* <0.001)	0.341 (*p* <0.001)	0.240 (0.004)	0.298 (*p* <0.001)
SCWT C (s)	0.297 (*p* <0.001)	0.383 (*p* <0.001)	0.331 (*p* <0.001)	0.266 (0.002)	0.247 (0.004)
SDMT	−0.356 (*p* <0.001)	−0.333 (*p* <0.001)	−0.366 (*p* <0.001)	−0.266 (0.002)	−0.269 (0.001)
TMT A (s)	0.298 (*p* <0.001)	0.290 (0.001)	0.311 (*p* <0.001)	0.244 (0.004)	0.309 (*p* <0.001)
TMT B (s)	0.305 (*p* <0.001)	0.296 (*p* <0.001)	0.311 (*p* <0.001)	0.252 (0.003)	0.245 (0.004)

*Data are presented as Spearman's correlation coefficient (r value) and (p-value). RD, radial diffusivity; MD, mean diffusivity*.

### The Results of Rich Club Analysis

**Figure 5:**
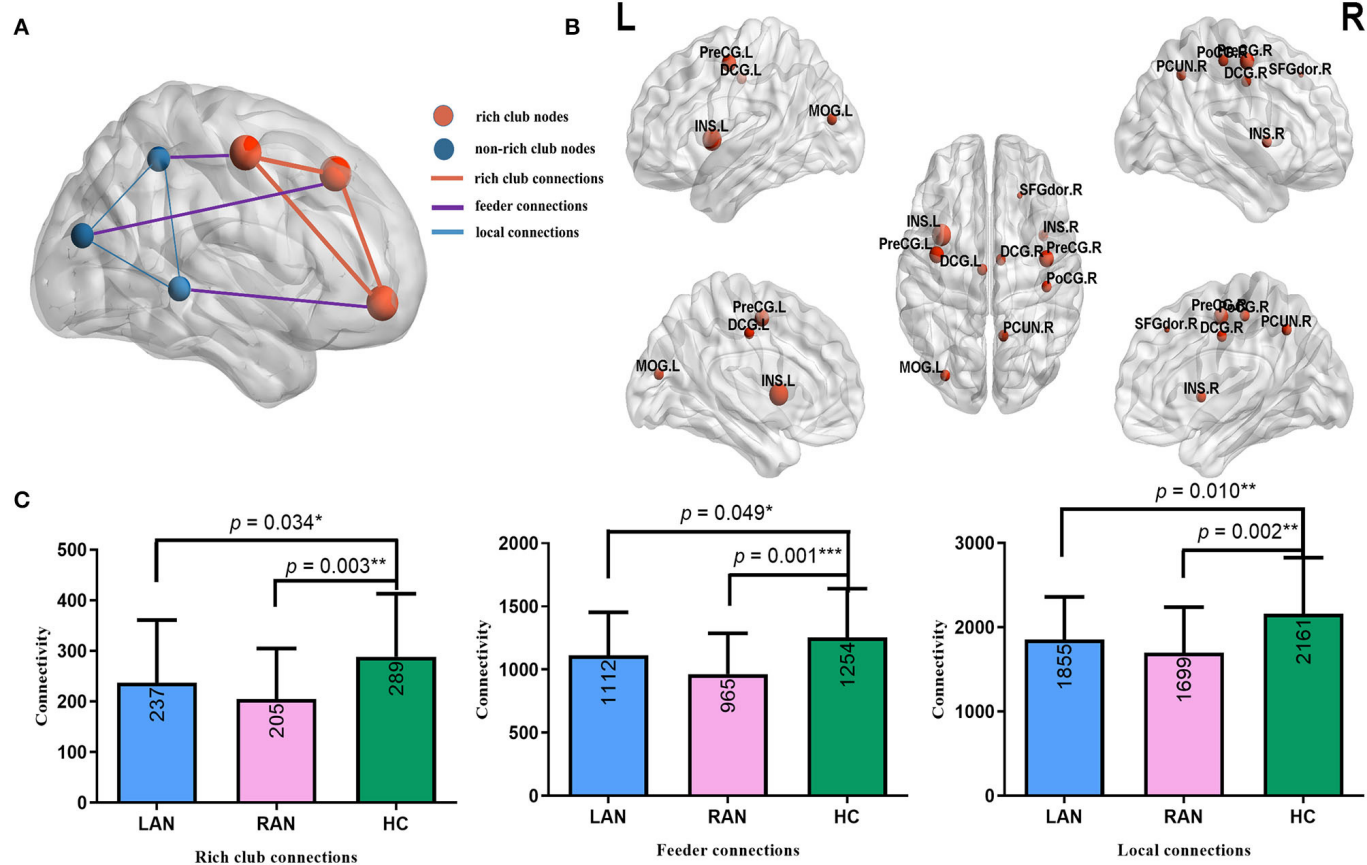
The related results of rich club analysis. **(A)** Schematic diagram of rich club analysis. Red nodes represent rich club nodes, and blue nodes represent non-rich club nodes. Sorting by degree centralities from large to small, the first 12% were taken as rich club nodes. Rich club connections (red lines) refer to the edges that link rich club nodes in the network; local connections (blue lines) refer to the edges that link non-rich club nodes; feeder connections (purple lines) refer to the edges that link rich club nodes and non-rich club nodes in the network. **(B)** In this study, 10 rich club nodes are shown, namely bilateral precentral gyrus, bilateral median cingulate and paracingulate gyri, bilateral insular, left middle occipital gyrus, right postcentral gyrus, right precuneus, and right dorsolateral superior frontal gyrus. **(C)** The comparison results of rich club analysis. Compared to the HC group, all the connections in patients with LAN and RAN decreased. *p < 0.05, **p ≤ 0.01, ***p ≤ 0.001. The numbers in the box represent mean values. Error bars represent mean ± SD.

Ten rich club nodes were as follows: bilateral precentral gyrus, bilateral median cingulate and paracingulate gyri, bilateral insular, left middle occipital gyrus, right postcentral gyrus, right precuneus, and right dorsolateral superior frontal gyrus, and the remaining 80 nodes were defined as non-rich club nodes. Compared with the HC group, all the connections in patients with LAN and RAN decreased, and the differences were statistically significant (see Figure 5).

## Discussion

The patients with LAN and RAN performed worse on MoCA, RAVLT, SCWT, SDMT, and TMT tests, revealing that patients with AN developed cognition declines such as attention, memory, executive function, information processing speed, and movement speed. Previous studies have established that cognitive decline occurs in patients with AN (Goebel and Mehdorn, 2018; Fan et al., 2020), however, little is known about the possible underlying mechanisms. In this study, we also found that general cognitive function, attention, executive function, memory, visuospatial and perception, movement speed, and information processing speed in patients with AN were significantly lower than those of normal people. Furthermore, we explored the possible mechanism using TBSS based on DTI data. We found FA, AD, RD, and MD values changed, and were related to cognitive impairment in patients with AN. DTI is used to evaluate the fiber direction and density, the integrity of myelin sheath, and the morphology of axons (Mori and van Zijl, 2002). Eigenvalues λ_1_, λ_2_, and λ_3_ are always used to describe the characteristics of white matter. FA values can be calculated and deduced according to λ_1_, λ_2_, and λ_3_. The AD is equal to λ_1_, and RD is equal to the average values of λ_2_ and λ_3_. Actually, FA is determined by AD and RD. FA is highly sensitive to microstructure changes, however, it is not specific to the types of changes. Theoretically, the decrease of FA may be caused by the decrease of AD or the increase of RD or the combination of both, and vice versa (Squarcina et al., 2017). AD may be affected by the diameter or density of white matter fibers, reflecting the integrity of the axon. And the increase in AD value reflects the decrease in the diameter or density of the axon (Rizk et al., 2017; Liu et al., 2018). RD mainly reflects myelin sheath integrity (Rizk et al., 2017; Liu et al., 2018), and the increase of RD value reflects the damage to myelin integrity, demyelination change, or poor myelination formation (Zheng et al., 2017). MD reflects the average degree of diffusion (Li et al., 2011). If white matter fiber is damaged, such as the decrease of cell membrane density, the limitation of water molecular dispersion decreases and it is easier to disperse, then the MD value will increase in theory (Hervé et al., 2005; Li et al., 2011). Therefore, it is suggested that multiple metrics (such as FA, MD, AD, and RD) should be used at the same time to better reflect the changes in white matter microstructure.

We found that compared with the HC group, the FA value of the minor forceps decreased. The corpus callosum transmits information about learning and involves executive function and memory (Voineskos et al., 2012). Tomimoto et al. (2004) found that the volume and FA of corpus callosum decreased in patients with vascular dementia. Kontis et al. (2009) demonstrated that the decrease of FA in the corpus callosum of premature infants is linked to poor language learning in adulthood. We also found that the decrease of FA in minor forceps was related to the decline of general cognitive function, attention, memory, executive control, and visuospatial and visuoperceptual abilities (see Table 2). To our knowledge, this is the first time to report this finding in patients with AN. Thus, we consider FA in minor forceps can be used as a neurobiological marker of cognitive impairment in patients with AN. To our surprise, the FA values in the bilateral corticospinal tract and left superior longitudinal fasciculus were higher than those in the HC group, so we paid special attention to the reasons AD and RD caused the increase of FA on these fiber bundles. In patients with AN, the AD values of the left superior longitudinal fasciculus and the right corticospinal tract were increased, and the RD values were normal. The increase in AD value may be caused by the decrease in fiber diameter or density, which reflects the destruction of axon integrity (Rizk et al., 2017; Liu et al., 2018; Jütten et al., 2019). No significant change in RD indicates that there is no significant change in myelin sheath in patients with AN. therefore, we believed that the increase of FA value in bilateral corticospinal tract and left superior longitudinal fasciculus in patients with AN was affected by the increase of AD value of fiber bundle, which essentially reflected the destruction of axonal integrity of corticospinal tract and left superior longitudinal fasciculus, rather than the increase of FA caused by myelin thickening caused by compensatory remodeling. Therefore, we chose the AD values of the corticospinal tract and the left superior longitudinal fasciculus instead of FA values to analyze the correlation with the cognitive scale.

The superior longitudinal fasciculus connects the frontotemporal parietal cortex, mainly involves the regulation of movement, working memory, attention, and language, and plays an important role in executive control, emotion regulation, and cognitive process (Wang et al., 2016; Zhang et al., 2016). In our study, the increased AD value of bilateral superior longitudinal fasciculus showed the destruction of axonal integrity and was associated with the decline of general cognitive function, memory, attention, language, and executive control ability. The corticospinal tract transmits motor information and participates in speech processing, and its damage is often related to decreased memory (Grahn et al., 2009) and visuospatial impairment (Su et al., 2007). Consistent with the previous report, we also found that the increased AD of the corticospinal tract was linked to the decrease of MOCA, visuospatial and executive, language fluency task, and RAVLT. The increase of RD in bilateral anterior thalamic radiations revealed the demyelination of white matter. The anterior thalamus radiation originates the medial thalamic nuclei and passes through the anterior limb of the internal capsule, projects to the frontal lobe, and the thalamic nucleus is complex and has fibrous connections with the hippocampus, striatum, and frontal cortex, which is related to cognitive function, memory and emotional regulation (Biesbroek et al., 2013; Spalletta et al., 2013). Therefore, the damage to the microstructure of anterior thalamic radiations may lead to the decline of related cognitive function. Our study also confirmed that anterior thalamic radiation was related to the decline of general cognitive function, memory, attention, and executive control in patients with AN, which was consistent with the reported literature (Biesbroek et al., 2013; Spalletta et al., 2013). The right inferior frontal-occipital fasciculus connects the frontal lobe and the temporal-occipital lobe, which may be linked to auditory-visual synesthesia, and also plays a major structural connection in the ventral attention network (Umarova et al., 2010), which is consistent with the conclusion that the MD value of the inferior frontal-occipital fasciculus was negatively correlated with the attention score (*r* = 0.376, *p* < 0.001) in our study. The frontal lobe is an important brain area of executive function, including mainly fiber bundles such as superior longitudinal fasciculus, corpus callosum, inferior frontal-occipital fasciculus, and inferior longitudinal fasciculus, which can lead to executive dysfunction. It has been found that the decrease of executive function in patients with white matter lesions is associated with the damage of white matter fiber in the frontal lobe, which may be linked to the disconnection of the executive loop in the frontal lobe (Munoz, 2006; Li et al., 2007). Correlation analysis in our study showed that the damage to the superior longitudinal fasciculus, corpus callosum, and inferior fronto-occipital fasciculus was related to the decrease in executive function in patients, which was consistent with the conclusions of previous studies (see Tables 2, 3).

Previous studies used ROI to focus on the microscopic changes of white matter fiber in the auditory pathway. In this study, no obvious changes were found in the auditory pathway. TBSS may not be as sensitive as ROI in the subtle structure, but ROI only pays attention to some areas of interest, while TBSS can explore the voxel level of the whole brain and find abnormal alterations that the ROI method is difficult to find.

We found that the rich club nodes of healthy people were mainly located in the DMN, sensorimotor network, attention network, salience network, and executive control network, which was consistent with the previous study (Li et al., 2021). The precentral gyrus belongs to the sensorimotor network. Studies have validated that the sensorimotor system not only initiates and regulates the sensation and movement of the body but also plays an important role in speech processing such as vocabulary, phonetics, sentences, and chapter processing (Fischer and Zwaan, 2008). The dorsolateral superior frontal gyrus is involved in memory execution, and the premotor area and prefrontal lobe are related to spatial memory (Smith et al., 1996). The key brain regions for retrieving memory include the right frontal lobe, anterior cingulate gyrus, parietal lobe, and thalamus (Nyberg et al., 1996). The insular mainly plays a key role in the switching between cognitive function-related networks (Seeley et al., 2007; Sidlauskaite et al., 2016). The precuneus is associated with episodic memory retrieval (Buckner and DiNicola, 2019). Therefore, these rich club regions involving memory, attention, and executive control were closely related to cognition. However, the rich club connections in patients with LAN and RAN were lower than those in the HC group, which were closely related to the decline in memory, attention, and executive control ability of patients. In the patients with LAN and RAN, the rich club connections, feeder connections, and local connections were all decreased, showing that the connections of the whole brain network in patients with AN had changed, which may lead to cognitive dysfunction.

However, this article still has the following weakness: (1) The study is a cross-sectional study that failed to follow up on the changes in cognitive function with the progress of the disease and the development of hearing level. (2) This study failed to follow up on whether the cognition recovered after hearing improvement in patients with AN. (3) Although TBSS is superior to other methods in the present study, it is still relatively new and needs additional comment. For example, the process assumes that in the white matter tract, the anisotropy value is the largest at the center of the tract, and gradually decreases away from the center. However, the assumption is not always correct. In several areas where two or more areas diverge or converge, more complex FA projection techniques are required (Smith et al., 2006). (4) It may need multimodal MRI (e.g., Morphological, Structural, and Functional Networks) to validate the results (Xu et al., 2021).

## Conclusion

In this study, we found that the cognitive function decreased in patients with AN, and we explored the underlying possible mechanism. The white matter showed extensive damage in patients with AN, which was related to the decline of general cognitive function, attention, memory, and executive function. At the same time, we found for the first time that the decline of cognitive function was related to the decrease of FA in minor forceps, which can be used as a neurobiological marker of cognitive impairment in patients with AN. The connections between the rich club brain regions involved in memory, language, attention, executive control, and other functions are decreased, leading to a decline in corresponding cognitive functions. The occurrence of cognition impairment is common in patients with AN. Including neuropsychological evaluation in the routine clinical assessment and appropriate treatment may strengthen clinical management and improve the quality of life of patients.

## Data Availability Statement

The raw data supporting the conclusions of this article will be made available by the authors, without undue reservation.

## Ethics Statement

The studies involving human participants were reviewed and approved by Ethics Committee of Nanchong Central Hospital. The patients/participants provided their written informed consent to participate in this study. Written informed consent was obtained from the individual(s) for the publication of any potentially identifiable images or data included in this article.

## Author Contributions

XD, LizL, and JL designed the study and collected the data. XD and LihL analyzed the data. XD wrote the paper. HF, LihL, and XH drafted the article. All authors contributed to the article and approved the submitted version.

## Funding

This work was supported by Nanchong Science and Technology Bureau (Grant Nos. 19SXHZ0273 and 20YFZJ0115), Nanchong Social Science Federation (Grant No. NC21B188), Sichuan Province Medical Youth Innovative Research Project Program (Grant No. Q21029), Primary Health Development Research Center of Sichuan Province (Grant No. SWFZ20-C-069), and Special Funding for Postdoctoral Research Projects of Chongqing.

## Conflict of Interest

The authors declare that the research was conducted in the absence of any commercial or financial relationships that could be construed as a potential conflict of interest.

## Publisher's Note

All claims expressed in this article are solely those of the authors and do not necessarily represent those of their affiliated organizations, or those of the publisher, the editors and the reviewers. Any product that may be evaluated in this article, or claim that may be made by its manufacturer, is not guaranteed or endorsed by the publisher.
