# Supplementary material for: Research on the Mechanism of Cognitive Decline in Patients With Acoustic Neuroma
Source: Front Neurosci. 2022 Jul 4;16:933825. doi: 10.3389/fnins.2022.933825 (PMC9289464; doi:10.3389/fnins.2022.933825)
Supplement: Supplementary file 1 [file Table_1.DOCX]

**Supplementary Table 1 Comparison of MoCA among patients with different grades of AN and HC group**

|  | Koos 1 | Koos 2 | Koos 3 | Koos 4 |
| --- | --- | --- | --- | --- |
| Visuospatial executive | N/A | -2.919(0.004) ^**^ | -4.371(*p* < 0.001) ^***^ | -5.552(*p* < 0.001) ^***^ |
| Naming | N/A | -0.039(0.969) | -1.422(0.155) | -2.355(0.019) ^*^ |
| Attention | N/A | -3.366(0.001) | -3.322(0.001) ^***^ | -4.576(*p* < 0.001) ^***^ |
| Language | N/A | -1.100(0.271) | -3.778(*p* < 0.001) ^***^ | -3.429(0.001) ^***^ |
| Language: Sentence repetition | N/A | -0.692(0.489) | -3.324(0.001) ^***^ | -3.048(0.002) ^**^ |
| Language: fluency task | N/A | -2.951(0.003) ^**^ | -4.061(*p* < 0.001) ^***^ | -3.680(*p* < 0.001) ^***^ |
| Abstract thinking | N/A | -0.123(0.902) | -2.081(0.037) ^*^ | -3.381(0.001) ^***^ |
| Delayed recall | N/A | -2.483(0.013) ^*^ | -1.804(0.071) | -3.512(*p* < 0.001) ^***^ |
| Orientation | N/A | -2.642(0.008) ^**^ | -2.560(0.010) ^**^ | -3.327(0.001) ^***^ |
| Moca scores | N/A | -2.652(0.008) ^**^ | -3.846(*p* < 0.001) ^***^ | -5.527(*p* < 0.001) ^***^ |

Data are presented as Z values (*p* values). Z and *p* values were obtained by Mann-Whitney U nonparametric test. *, *p* < 0.05; **, *p* ≤ 0.01; ***, *p* ≤ 0.001; N/A: not available; MoCA: Montreal cognitive assessment.

|  | Koos 1 | Koos 2 | Koos 3 | Koos 4 |
| --- | --- | --- | --- | --- |
| MoCA | N/A | -2.652(0.008) ^**^ | -3.846(*p* < 0.001) ^***^ | -5.527(*p* < 0.001) ^***^ |
| RAVLT  (immediate recall) | N/A | -2.804(0.005) ^**^ | -3.476(0.001) ^***^ | -5.044(*p* < 0.001) ^***^ |
| RAVLT  (delay recall) | N/A | -1.966(0.053) | -3.457(0.001) ^***^ | -6.035(*p* < 0.001) ^***^ |
| SCWT A (s) | N/A | -0.626(0.531) | -1.746(0.081) | -2.905(0.004) ^**^ |
| SCWT B (s) | N/A | -0.183(0.855) | -2.758(0.006) ^**^ | -4.199(*p* < 0.001) ^***^ |
| SCWT C (s) | N/A | -0.921(0.357) | -3.630(*p* < 0.001) ^***^ | -4.993(*p* < 0.001) ^***^ |
| SDMT | N/A | -0.807(0.419) | -2.560(0.010) ^**^ | -3.794(*p* < 0.001) ^***^ |
| TMT A (s) | N/A | -1.410(0.159) | -2.597(0.009) ^**^ | -3.978(*p* < 0.001) ^***^ |
| TMT B (s) | N/A | -0.339(0.735) | -2.003(0.045) ^*^ | -4.570(*p* < 0.001) ^***^ |

**Supplementary Table 2 Comparison of cognitive function among patients with different grades of AN and HC group**

Data are presented as Z values (*p* values). Z and p values were obtained by Mann-Whitney U nonparametric test. *, *p* < 0.05; **, *p* ≤ 0.01; ***, *p* ≤ 0.001. N/A: not available.

**Supplementary Table 3 Comparison of MoCA among patients with different degrees of hearing loss and HC group**

|  | Normal hearing (n = 5) | Mild loss  (n = 12) | Moderate loss  (n = 9) | Severe loss  (n = 13) | Profound loss  (n = 10) |
| --- | --- | --- | --- | --- | --- |
| Visuospatial executive | -1.607(0.132) | -2.723(0.006) ^**^ | -3.159(0.002) ^**^ | -4.170 (*p* < 0.001) ^***^ | -3.845(*p* < 0.001) ^***^ |
| Naming | -0.741(0.556) | -1.151(0.250) | -0.202(0.840) | -2.817(0.005) ^**^ | -1.159(0.246) |
| Attention | -1.022(0.488) | -1.583(0.113) | -1.713(0.087) | -4.174 (*p* < 0.001) ^***^ | -4.482(*p* < 0.001) ^***^ |
| Language | -0.783(0.475) | -2.181(0.029) ^*^ | -1.362(0.173) | -2.591(0.010) ^**^ | -2.797(0.005) ^**^ |
| Language: Sentence repetition | -0.267(0.437) | -1.810(0.070) | -1.181(0.238) | -2.398(0.017) ^*^ | -2.676(0.007) ^**^ |
| Language: fluency task | -0.267(0.959) | -2.582(0.010) ^**^ | -3.053(0.002) ^**^ | -3.327(0.001) ^***^ | -2.873(0.004) ^**^ |
| Abstract thinking | -0.413(0.718) | -0.058(0.954) | -2.265(0.024) ^*^ | -2.860(0.004) ^**^ | -0.690(0.490) |
| Delayed recall | -1.613(0.116) | -0.870(0.384) | -0.165(0.869) | -3.027(0.002) ^**^ | -2.782(0.005) ^**^ |
| Orientation | -0.737(0.718) | -0.629(0.529) | -1.886(0.059) | -3.236(0.001) ^***^ | -4.071(*p* < 0.001) ^***^ |
| Moca scores | -1.536(0.132) | -2.016(0.044) ^*^ | -2.325(0.020) ^*^ | -3.961(*p* < 0.001) ^***^ | -3.651(*p* < 0.001) ^***^ |

Data are presented as Z values (*p* values). *Z* and p values were obtained by Mann-Whitney U nonparametric test. Normal hearing: PTA < 25dB HL; mild loss: PTA 26-40 dB HL; moderate loss: PTA 41-60 dB HL; severe loss: PTA 61-80 dB HL; profound loss: PTA > 81 dB HL. *, *p* < 0.05; **, *p*≤ 0.01; ***, *p* ≤ 0.001. N/A: not available. Due to the impact of COVID-19, only 49 patients in total have undergone audiometric tests.

**Supplementary Table 4 Comparison of cognitive function among patients with different degrees of hearing loss and HC group.**

|  | Normal hearing (n = 5) | Mild loss  (n = 12) | Moderate loss  (n = 9) | Severe loss  (n = 13) | Profound loss  (n = 10) |
| --- | --- | --- | --- | --- | --- |
| MoCA | -1.536(0.132) | -2.016(0.044) * | -2.325(0.020) * | -3.961(*p* < 0.001) *** | -3.651(*p* < 0.001) *** |
| RAVLT  (immediate recall) | -0.893(0.389) | -2.869(0.004) ** | -1.359(0.174) | -3.391(0.001) *** | -3.858(*p* < 0.001) *** |
| RAVLT  (delay recall) | -1.228(0.230) | -2.117(0.034) * | -1.058(0.293) ^a^ | -3.321(0.001) ^a^*** | -4.177(*p* < 0.001) ^a^*** |
| SCWT A (s) | -1.155(0.263) | -0.666(0.505) | -0.157(0.875) | -1.904(0.057) | -2.367(0.018) * |
| SCWT B (s) | -0.648(0.528) | -0.774(0.439) | -0.448(0.654) | -3.378(0.001) *** | -3.165(0.002) ** |
| SCWT C (s) | -0.680(0.515) | -2.060(0.039) * | -1.837(0.066) | -2.572(0.010) ** | -3.274(0.001) *** |
| SDMT | -1.349(0.183) | -0.794(0.427) | -1.034(0.301) | -2.664(0.008) ** | -3.006(0.003) ** |
| TMT A (s) | -0.754(0.462) | -1.116(0.265) | -1.351(0.177) | -3.265(0.001) *** | -3.202(0.001) *** |
| TMT B (s) | -0.074(0.943) | -1.995(0.046) * | -0.301(0.763) | -3.104(0.002) ** | -2.431(0.015) * |

Data are presented as T or Z values (*p* values). *T* and ^a^ *p* values were obtained by t-test. Z values were obtained by Mann-Whitney U nonparametric test. Normal hearing: PTA < 25dB HL; mild loss: PTA 26-40 dB HL; moderate loss: PTA 41-60 dB HL; severe loss: PTA 61-80 dB HL; profound loss: PTA > 81 dB HL. *, *p* < 0.05; **, *p* ≤ 0.01; ***, *p* ≤ 0.001. N/A: not available.
